# Somatic DNA Damage Response and Homologous Repair Gene Alterations and Its Association With Tumor Variant Burden in Breast Cancer Patients With Occupational Exposure to Pesticides

**DOI:** 10.3389/fonc.2022.904813

**Published:** 2022-07-08

**Authors:** Thalita Basso Scandolara, Sara Ferreira Valle, Cristiane Esteves, Nicole de Miranda Scherer, Elvismary Molina de Armas, Carolina Furtado, Renan Gomes, Mariana Boroni, Hellen dos Santos Jaques, Fernanda Mara Alves, Daniel Rech, Carolina Panis, Cibele Rodrigues Bonvicino

**Affiliations:** ^1^Department of Genetics, Biology Institute, Universidade Federal do Rio de Janeiro, Rio de Janeiro, Brazil; ^2^Bioinformatics and Computational Biology Laboratory, Instituto Nacional de Câncer José Alencar Gomes da Silva (INCA), Rio de Janeiro, Brazil; ^3^Department of Informatics, Pontificia Universidade Católica (PUC)-Rio, Rio de Janeiro, Brazil; ^4^Division of Genetics, Instituto Nacional de Câncer José Alencar Gomes da Silva (INCA), Rio de Janeiro, Brazil; ^5^Laboratory of Tumor Biology, State University of West Paraná, Francisco Beltrão, Brazil; ^6^Francisco Beltrão Cancer Hospital, Francisco Beltrão, Brazil

**Keywords:** breast cancer, pesticides, occupational exposure, mutational burden, somatic

## Abstract

Homologous recombination is a crucial pathway that is specialized in repairing double-strand breaks; thus, alterations in genes of this pathway may lead to loss of genomic stability and cell growth suppression. Pesticide exposure potentially increases cancer risk through several mechanisms, such as the genotoxicity caused by chronic exposure, leading to gene alteration. To analyze this hypothesis, we investigated if breast cancer patients exposed to pesticides present a different mutational pattern in genes related to homologous recombination (*BRCA1*, *BRCA2*, *PALB2*, and *RAD51D*) and damage-response (*TP53*) concerning unexposed patients. We performed multiplex PCR-based assays and next-generation sequencing (NGS) of all coding regions and flanking splicing sites of *BRCA1*, *BRCA2*, *PALB2*, *TP53*, and *RAD51D* in 158 unpaired tumor samples from breast cancer patients on MiSeq (Illumina) platform. We found that exposed patients had tumors with more pathogenic and likely pathogenic variants than unexposed patients (p = 0.017). In general, tumors that harbored a pathogenic or likely pathogenic variant had a higher mutational burden (p < 0.001). We also observed that breast cancer patients exposed to pesticides had a higher mutational burden when diagnosed before 50 years old (p = 0.00978) and/or when carrying *BRCA1* (p = 0.0138), *BRCA2* (p = 0.0366), and/or *PALB2* (p = 0.00058) variants, a result not found in the unexposed group. Our results show that pesticide exposure impacts the tumor mutational landscape and could be associated with the carcinogenesis process, therapy response, and disease progression. Further studies should increase the observation period in exposed patients to better evaluate the impact of these findings.

## Introduction

Brazil is one of the leading agricultural pesticide-consuming countries in the world (1). The extensive use of pesticides raises concerns about human health (2). Since 2008, Brazil has become the world’s top pesticide importer, with more than 1,400 formulations authorized by the government legislation (3). Only 3.5% of the total pesticides authorized in Brazil are approved in other countries due to their high toxicity and their recognized carcinogenic potential. In this context, a total of 52 pesticides used in Brazil are classified as “probable” or “possible” carcinogens for humans, 16 had evidence suggestive of the carcinogenic potential for humans, and eight had insufficient information about the carcinogenic potential for humans (4). Although the use of pesticides has been widespread in Brazil since the 1960s, the exact data concerning pesticide consumption from small-scale farmers are scarce (5–7). Improper pesticide application associated with farmers’ limited knowledge regarding its harmful effects and poor adherence to safety precautions, such as the correct use of personal protective equipment, represent a considerable health risk for chronically exposed populations (8, 9).

Pesticide exposure has the potential to increase cancer risk through several mechanisms, including oxidative stress generation, changes in adhesion molecules, acetylcholinesterase inhibition, endocrine disruption, and contribution to genomic instability (5), which are known hallmarks of cancer (10). Double-strand breaks can be a threat to genomic stability. Multiple DNA repair mechanisms are available to counteract its deleterious effects, as failure to repair double-strand breaks can result in chromosome aberrations, apoptosis, and oncogenesis (11). In this regard, homologous recombination is a crucial pathway specialized in repairing double-strand breaks that occur mainly during DNA replication and through cell damage (12). Specific hereditary cancer predisposition syndromes, such as hereditary breast and ovarian cancer (HBOC) and Fanconi anemia, are associated with germline mutations in *BRCA1/2* and *PALB2* genes, respectively (13). These genes are essential for homologous recombination, and alterations in genes involved in this pathway are closely associated with carcinogenic features such as high mutational burden, which is caused by the accumulation of unrepaired DNA damage (11, 13). The loss of function of tumor suppressor genes in tumors, such as *TP53* gene, has shown that DNA damage response pathways must be downregulated to guarantee cell proliferation and for cells to avoid apoptosis (14). Thus, tumoral cells often harbor alterations in genes responsible for these DNA damage response pathways, which may lead to loss of genomic stability and cell growth suppression (10, 15).

It is suggested that only 5%–10% of breast cancer cases are hereditary; the remaining ~90% are associated, to a greater or lesser extent, with environmental factors that result in the occurrence and accumulation of somatic and epigenetic alterations during a person’s life (16). Thus, lifestyle and environmental conditions have a significant impact on breast cancer risk. Evaluation of mutational patterns related to DNA damage and repair processes in cancer revealed that several signatures could be associated with carcinogen exposures and defects in DNA maintenance pathways, such as specific base transversions found in smoking-associated lung cancer and caused by tobacco exposure (17). While factors such as obesity, physical activity, and consumption of tobacco and alcohol are known to be associated with breast cancer risk (18–20), the potential roles of environmental exposure to pesticides in breast cancer development are not well understood. Even so, the lack of high-quality and significant evidence, concerning the relationship between pesticide exposure and cancer risk from epidemiological studies, makes it challenging to infer causality. While most studies indicate a trend toward increased risk, only a few are statistically significant, as reviewed elsewhere (21). Although inconsistent results are found in the literature regarding pesticide exposure and breast cancer risk, it is suggested that hormone-positive breast cancer could have an increased association with pesticide exposure (22).

Considering the genotoxicity potential of pesticides, and that the homologous repair pathway may be an essential mechanism for DNA maintenance under such circumstances, we investigated if the profile of acquired genetic mutations in breast tumors is related to patients’ occupational exposure. We hypothesized that it might be associated with increased genomic instability and truncating variants, especially in genes responsible for DNA damage response and known tumor suppressor genes, which could directly impair cell response against genotoxicity and favor oncogenesis and/or disease progression.

## Materials and Methods

### Study Population

A total of 541 patients were enrolled in this study between January 2015 and September 2019. They were managed at Francisco Beltrão Cancer Hospital (Ceonc), which assists 27 municipalities in Paraná state, Brazil. After patients signed consent forms, tumor samples were collected consecutively with no selection bias during the diagnostic breast cancer biopsy surgery. The study was reviewed and approved by the Ethics Committees of the State University of West Paraná, under the number CAAE 35524814.4.0000.0107, and was performed following the Declaration of Helsinki.

**Figure 1:**
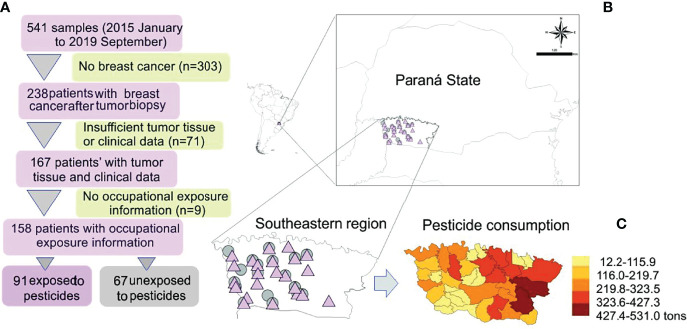
**(A)** Study design and map showing the sample distribution according to exposure. **(B)** Amplified map of Brazilian Paraná State with symbols representing exposed (pink triangle) and unexposed (gray circle) patients’ localities. **(C)** Detail of pesticide consumption in the study area; modified from Gaboardi et al. (24).

All enrolled patients were invited to answer a questionnaire with 61 questions about their current and past occupational history (23). Among the 167 women diagnosed with breast cancer, 158 were eligible for this study. Based on their answers, the study population was categorized as occupationally exposed or not to pesticides. The study was composed of 91 exposed patients and 67 unexposed to pesticides (**Figure 1**).

### DNA Extraction and Clinicopathological Data Obtention

Tumor samples were stored under refrigeration at −20°C until genomic DNA extraction with QIAamp DNeasy Blood & Tissue kit (QIAGEN, Valencia, CA, USA), following the manufacturer’s recommendations. All clinicopathological variables were collected from medical records. Patients were categorized based on their age, tumor grade, hormonal receptor status (estrogen receptor (ER), progesterone receptor (PR), and human epidermal growth factor receptor 2 (HER2)), molecular subtype, lymph nodal invasion, tumor size, distant metastasis, menopausal status, chemoresistance and/or tumor persistence, disease relapse, and survival status. For HER2 status, an immunohistochemistry score of 0 and 1+ was considered negative, and 2+ and 3+ were considered positive, where fluorescence *in situ* hybridization (FISH) further confirmed 2+. Early disease onset was categorized as <50 years old and late disease onset as ≥50 years old.

### Sample Preparation and Next-Generation Sequencing of Unpaired Tumor

Multiplex PCR-based assays were performed on 158 unpaired tumor samples, designed to cover the entire coding regions and flanking splicing sites of *BRCA1*, *BRCA2*, *PALB2*, *TP53*, and *RAD51D* (25). Tumor DNA sequencing was prepared with Nextera XT DNA Kit (Illumina, San Diego, CA, USA) and performed in three independent runs, using paired-end methodology on MiSeq Genome Analyzer (Illumina), with up to 150 bp of reading length.

### Somatic Variants Calling and Enrichment in Unpaired Tumor Samples

Base quality score recalibration, indel realignment, and variant calling were performed following the Genome Analysis Toolkit v4.1 (GATK, Broad Institute) best practice (https://software.broadinstitute.org/gatk/best-practices) (26). Somatic variants were called by Mutect2 from GATK, using default parameters for unpaired tumor samples. Somatic variants were functionally annotated using GATK Funcotator (27) and the Ensembl Variant Effect Predictor (VEP) (28). To enrich in true positives, all the following parameters were applied: i) removal of single-nucleotide polymorphisms (SNPs) observed in the 1000 Genomes Project, the Exome Aggregation Consortium (ExAC), observed in common populations with minor allele frequency  >0.5%, according to Genome Aggregation Database (gnomAD); ii) support from ≥20 reads in the tumor; iii) a variant allele fraction (VAF) of ≥0.02; iv) support from reads mapped to both strands; v) synonymous variants were excluded.

### Tumor Mutational Burden Analysis

To explore the mutational landscape according to pesticide exposure, somatic variant data were processed and analyzed using the R programming language (version 4.0.2) with the “maftools” package (29). Tumor mutational burden (TMB) was measured by the total number of somatic non-synonymous variants in the target region by the total size of the target region per mega-base. Breast cancer patients were then separated into low- and high-TMB groups using the median value (**Supplementary Table I**). To analyze the correlations with clinicopathological features of patients with breast cancer, the mutational burden data were merged with corresponding clinical information. The Wilcoxon rank-sum test (30) and Fisher’s exact test (31) were used for comparisons between two groups of clinical variables, with p < 0.05 considered significant.

### Classification of Somatic Variants

All intronic, 5′Flank, 3′UTR, and silent variants were excluded. The pathogenicity of variants was determined predominantly based on the clinical data reported in ClinVar (http://www.ncbi.nlm.nih.gov/clinvar/), including benign, likely benign, variant of uncertain significance (VUS), likely pathogenic, and pathogenic. All nonsense and frameshift mutations not registered in the ClinVar, Catalogue of Somatic Mutations in Cancer (COSMIC), or International Agency for Research on Cancer (IARC) database were determined as pathogenic, according to the American College of Medical Genetics and Genomics (ACMG) standard terminology (32). Novel variants in a splice site that were not yet described were determined as likely pathogenic if there were known pathogenic variants in the same splice site already registered in ClinVar. Variants registered in ClinVar as “conflicting interpretations of pathogenicity” were classified as benign if “benign” or “likely benign” reports were predominant and were classified as likely pathogenic if “pathogenic” or “likely pathogenic” reports were predominant. If there was no meaningful information on the pathogenicity of variants in ClinVar, the COSMIC (http://cancer.sanger.ac.uk/cosmic) database was referred to. For *TP53* variants, a functional classification based on its translational activity in the IARC *TP53* Database (http://p53.iarc.fr/) was also referred to. All variants classified as “benign” and “likely benign” were lumped together into benign.

### Statistical Analysis

Statistical models included subjects with complete data on the specific environmental variable of interest and the adjustment variables. All statistical analyses and the visualizations were performed in the R programming language (version 4.0.2). The Shapiro–Wilk test (33) was applied to determine the normality of the data. Fisher’s exact test was used to compare frequencies between groups. Fisher’s exact test for co-occurrence analysis between mutated genes and clinical features was performed. For continuous variables and group comparisons, the Mann–Whitney U test (34) was performed. p-Values <0.05 were considered statistically significant.

## Results

Clinical and demographic characteristics of 158 women included in this study were observed (**Table 1**). The median age of diagnosis was 56.8 years (31–86 years). Statistically significant data showed that exposed patients presented a higher frequency of ER-negative (p = 0.0161) and PR-negative (p = 0.0014) tumors than did unexposed patients. Tumor samples were sequenced with a high depth, with a mean coverage of samples of 520× (102× to 1,068×). After processing and filtering steps for somatic variant identification, of 158 samples, 120 harbored 258 variants that were classified as missense, frameshift, nonsense, or splice-site variants and were distributed in *BRCA1*, *BRCA2*, *PALB2*, *TP53*, and *RAD51D* genes. We found 78 samples with variants in *BRCA2* (65%), 47 in *PALB2* (39.1%), 36 in *BRCA1* (30%), 27 in *TP53* (22.5%), and 13 in *RAD51D* (10.8%). The proportion of missense variants was the highest among other mutation types (~88.4%), and all variants were found in heterozygosity.

**Table 1:** Comparison of selected characteristics of the 158 breast cancer patients exposed and unexposed to pesticides.

Variable		Exposed (n = 68)	Unexposed (n = 52)	p-Value
**Age at diagnosis (years, mean ± SD)**		57.66 ± 14.5	55.63 ± 11.7	
**Tumor grade**	I/II	44 (64.7%)	40 (76.9%)	0.06
	III	24 (35.3%)	12 (23.1%)	
**ER status**	Positive	40 (58.8%)	39 (75%)	**0.0161**
	Negative	28 (41.2%)	13 (25%)	
**PR status**	Positive	26 (38.2%)	31 (59.6%)	**0.0014**
	Negative	42 (61.8%)	20 (38.4%)	
	Not informed	–	1 (1%)	
**HER2 status**	Positive	9 (13.2%)	7 (13.5%)	>0.9999
	Negative	59 (86.8%)	45 (86.5%)	
**Molecular subtype**	Luminal A	19 (28%)	19 (36.5%)	0.2899
	Luminal B	21 (30.9%)	17 (32.7%)	
	HER2+	9 (13.1%)	7 (13.5%)	
	Triple negative	19 (28%)	9 (17.3%)	
**Lymph node metastasis**	Positive	30 (44.1%)	23 (44.2%)	>0.9999
	Negative	38 (55.9%)	29 (55.8%)	
**Tumor size**	<2 cm	22 (32.3%)	20 (38.5%)	0.5885
	2–5 cm	35 (51.5%)	23 (44.2%)	
	>5 cm	11 (16.2%)	9 (17.3%)	
**Distant metastasis**	Yes	10 (14.7%)	8 (15.4%)	>0.9999
	No	58 (85.3%)	44 (84.6%)	
**Menopausal status**	Premenopause	19 (28%)	16 (30.8%)	0.6418
	Postmenopause	49 (72%)	36 (69.2%)	
**Chemoresistance**	Positive	22 (32.3%)	14 (27%)	0.4382
	Negative	46 (67.7%)	38 (73%)	
**Disease relapse**	Yes	8 (11.3%)	9 (17.3%)	0.3153
	No	60 (88.2%)	43 (82.7%)	
**Survival status**	Alive	60 (88.2%)	46 (88.4%)	>0.9999
	Deceased	8 (11.3%)	6 (11.6%)	

The bold values represent significant statistical differences (Fisher’s exact test, p < 0.05).

ER, estrogen receptor; PR, progesterone receptor; HER2, human epidermal growth factor receptor 2.

### Variant Landscape of Breast Cancer Samples and Grouped According to Patients’ Pesticide Exposure

**Figure 2:**
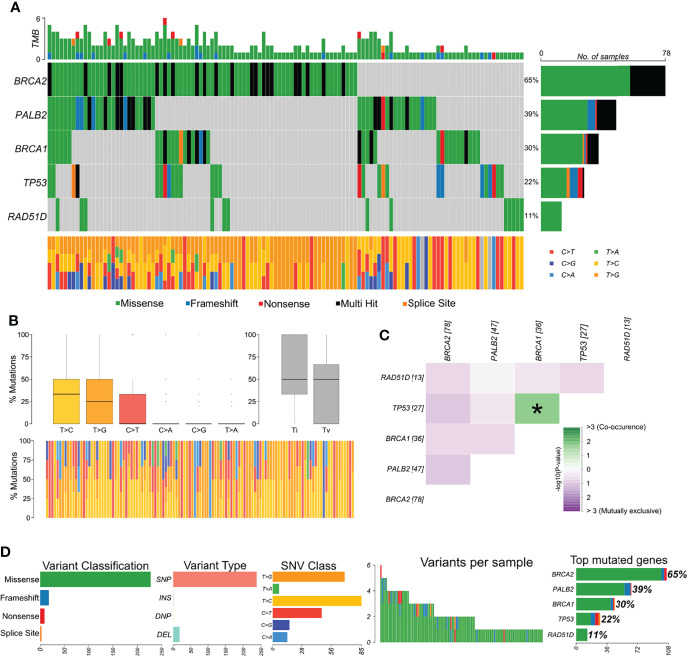
Genomic landscape and potential associations between mutations identified in tumor breast cancer samples. **(A)** Oncoplot showing the mutational profile of each gene in all tumor samples ordered by variant frequency. **(B)** Distribution of transitions and transversions in all tumor samples showing boxplot with overall summary of single-nucleotide variants (SNV) classified into six substitution classes, boxplot with distribution of SNVs classified into transitions (Ti) and transversions (Tv), and proportion of SNVs per sample classified into six substitution classes. **(C)** Co-occurrence or exclusive variant associations of the evaluated genes. **(D)** Panels with variant classification, type (SNP, single-nucleotide polymorphism; INS, insertion; DNP, double nucleotide polymorphism; DEL, deletion), SNV class, number of variants per sample, variant classification summary, and genes ordered by total number of variants. Color legend of panel A is the same for panels **(B, D)**.

The distribution of variants in all samples showed *BRCA2* as the most affected gene (**Figure 2A**). The most frequent variant type was SNPs/single-nucleotide variants (SNVs), and the most frequent nucleotide substitution was T>C, followed by T>G (**Figures 2B, C**). Approximately 75% of all variants identified by our study were classified as benign, 3.5% as likely pathogenic, 13.5% as pathogenic, 5% as VUS, and 3.5% have no classification in any database. Afterward, we explored whether variants were mutually exclusive or likely to co-occur (**Figure 2C**), and we observed the co-occurrence of variants in *BRCA1* and *TP53* in a significant manner (p < 0.05). In general, missense variants were the most frequent type (~88.5%), followed by frameshift (~7%), nonsense (3.5%), and splicing variants (1%) (**Figure 2D**).

**Figure 3:**
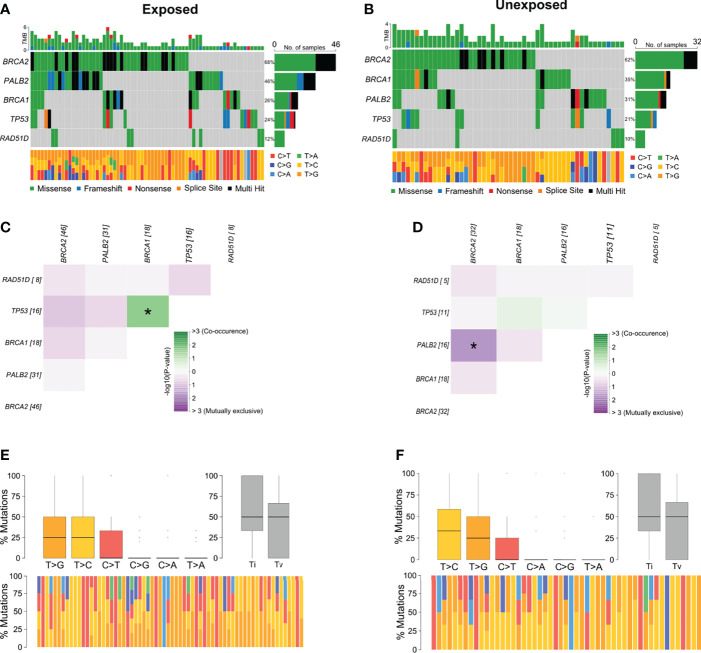
Genomic landscape and potential associations between mutations in tumor breast cancer samples grouped according to pesticide occupational exposure. **(A, B)** Oncoplots showing the mutational profile of each gene in groups exposed and unexposed to pesticides. **(C, D)** Mutational co-occurrence or exclusive associations between evaluated genes in **(C)** exposed and **(D)** unexposed groups. **(E, F)** Distribution of transitions and transversions in **(E)** exposed and **(F)** unexposed groups showing boxplot with overall summary of SNVs classified into six substitution classes, boxplot with distribution of SNVs classified into transitions (Ti) and transversions (Tv), and proportion of SNVs per sample classified into six substitution classes. Color legend of panels **(A, B)** is the same for panels **(E, F)**, respectively. The symbol * means statistical significance (p < 0.05).

Among breast cancer samples with variants detected, 56.6% (n = 68) belonged to patients with occupational exposure to pesticides whereas 43.3% (n = 52) to patients with no exposure. The most mutated genes in the exposed group were *BRCA2* and *PALB2* (**Figure 3A**) and in the unexposed group were *BRCA2* and *BRCA1* (**Figure 3B**). Concerning the co-interaction analysis, we observed a different correlation result among the exposed and unexposed groups (**Figures 3C, D**). Variants in *TP53* co-occur with *BRCA1* mutations in the exposed group (p < 0.05), while *PALB2* variants were found to occur in a mutually exclusive manner concerning *BRCA2* in the unexposed group (p < 0.05). Transversions T>G were higher in the exposed group than in the unexposed group, with more T>C transitions (**Figures 3E, F**).

### Frequency of Pathogenic, Likely Pathogenic, and Uncertain Significance Variants

We identified 28 pathogenic, 10 likely pathogenic, and 12 VUS variants (**Table 2**; **Supplementary Figure 1**). All variants were identified in 47 tumor samples from different patients. Several variants were detected in more than one sample: a) the pathogenic variants p.M296fs* in *PALB2* (6 samples), p.C61G in *BRCA1* (2 samples), and p.E198* in *TP53* (2 samples); b) the likely pathogenic variant p.H193R in *TP53* (2 samples); c) the VUS p.S46C in *RAD51D* (2 samples). Concerning *TP53*, we found 26 variants classified as pathogenic and likely pathogenic on ClinVar, being 21 of them also predicted as pathogenic on COSMIC. Six *BRCA1* variants are classified as pathogenic on ClinVar, 21 as pathogenic on the COSMIC database, and only one ranked as pathogenic in both databases (*BRCA1* c.181T>G). Four *BRCA2* variants are pathogenic on ClinVar and six on COSMIC, without concordance among both databases. Eight *PALB2* variants were classified as pathogenic on ClinVar, 12 on COSMIC, and only one classified as pathogenic in both databases (*PALB2* c.1240C>T). We found two *RAD51D* classified as VUS on ClinVar, and one of them was predicted as pathogenic in COSMIC (*RAD51D* c.137C>G).

**Table 2:** List of variants classified as pathogenic, likely pathogenic, and VUS.

Gene	cDNA change	Protein change	Type	Mutated samples	Previously reported	Pesticide exposure
***BRCA1***	c.181T>G	p.C61G	SNP	2	Yes	Exposed/unexposed
***BRCA1***	c.3329delA	p.K1110fs	DEL	1	Yes	Exposed
***BRCA1***	c.3790_3797delAAGAATAG	p.K1264fs	DEL	1	No	Exposed
***BRCA1***	c.1687C>T	p.Q563*	SNP	1	Yes	Exposed
***BRCA1***	c.3765_3786delCACAGAGGAGAATTTATTATCA	p.T1256fs	DEL	1	No	Exposed
***BRCA1***	c.5129G>A	p.G1710E	SNP	1	Yes	Exposed
***BRCA1***	c.546G>T	p.L182F	SNP	1	Yes	Unexposed
***BRCA1***	c.1996C>G	p.L666V	SNP	1	Yes	Unexposed
***BRCA2***	c.2806_2809delAAAC	p.A938fs	DEL	1	Yes	Unexposed
***BRCA2***	c.7879A>T	p.I2627F	SNP	1	Yes	Exposed
***BRCA2***	c.5067delA	p.K1691fs	DEL	1	Yes	Exposed
***BRCA2***	c.1314delT	p.T441fs	DEL	1	Yes	Exposed
***BRCA2***	c.5687C>T	p.A1896V	SNP	1	Yes	Exposed
***BRCA2***	c.5096A>G	p.D1699G	SNP	1	Yes	Unexposed
***BRCA2***	c.670G>A	p.D224N	SNP	1	Yes	Unexposed
***BRCA2***	c.3562A>G	p.I1188V	SNP	1	Yes	Exposed
***PALB2***	c.1314delA	p.F440fs	DEL	1	Yes	Exposed
***PALB2***	c.886delA	p.M296fs	DEL	6	Yes	Exposed
***PALB2***	c.1240C>T	p.R414*	DEL	1	Yes	Unexposed
***PALB2***	c.2453T>C	p.F818S	SNP	1	Yes	Unexposed
***PALB2***	c.2201C>A	p.T734N	SNP	1	Yes	Unexposed
***PALB2***	c.2608G>A	p.V870I	SNP	1	Yes	Exposed
***TP53***	c.481delG	p.A161fs	DEL	1	No	Exposed
***TP53***	c.592G>T	p.E198*	SNP	2	Yes	Exposed
***TP53***	c.856G>A	p.E286K	SNP	1	Yes	Exposed
***TP53***	c.730G>A	p.G244S	SNP	1	Yes	Exposed
***TP53***	c.734G>A	p.G245D	SNP	1	Yes	Unexposed
***TP53***	c.578A>C	p.H193P	SNP	1	Yes	Unexposed
***TP53***	c.617delT	p.L206fs	DEL	1	No	Unexposed
***TP53***	c.736A>G	p.M246V	SNP	1	Yes	Exposed
***TP53***	c.454_466delCCGCCCGGCACCC	p.P152fs	DEL	1	Yes	Exposed
***TP53***	c.586C>T	p.R196*	SNP	1	Yes	Exposed
***TP53***	c.626_627delGA	p.R209fs	DEL	1	Yes	Exposed
***TP53***	c.637C>T	p.R213*	SNP	1	Yes	Exposed
***TP53***	c.742C>T	p.R248W	SNP	1	Yes	Unexposed
***TP53***	c.818G>A	p.R273H	SNP	1	Yes	Exposed
***TP53***	c.447delC	p.T150fs	DEL	1	No	Unexposed
***TP53***	c.517G>A	p.V173M	SNP	1	Yes	Exposed
***TP53***	c.796G>A	p.G266R	SNP	1	Yes	Exposed
***TP53***	c.535C>A	p.H179N	SNP	1	Yes	Unexposed
***TP53***	c.536A>G	p.H179R	SNP	1	Yes	Unexposed
***TP53***	c.578A>G	p.H193R	SNP	2	Yes	Exposed/unexposed
***TP53***	c.711G>T	p.M237I	SNP	1	Yes	Unexposed
***TP53***	c.832C>T	p.P278S	SNP	1	Yes	Exposed
***TP53***	c.614A>G	p.Y205C	SNP	1	Yes	Exposed
***TP53***	c.613T>C	p.Y205H	SNP	1	Yes	Unexposed
***TP53***	c.96+2T>CTGGT	Splice site	INS	1	No	Unexposed
***TP53***	c.559+1G>C	Splice site	SNP	1	No	Exposed
***RAD51D***	c.56T>C	p.L19P	SNP	1	Yes	Unexposed
***RAD51D***	c.137C>G	p.S46C	SNP	2	Yes	Exposed/unexposed

cDNA and protein change, variant type, and number of tumors harboring each variant in patients exposed and unexposed to pesticides.

VUS, variant of uncertain significance; SNP, single-nucleotide polymorphism; INS, insertion; DEL, deletion.

**Figure 4:**
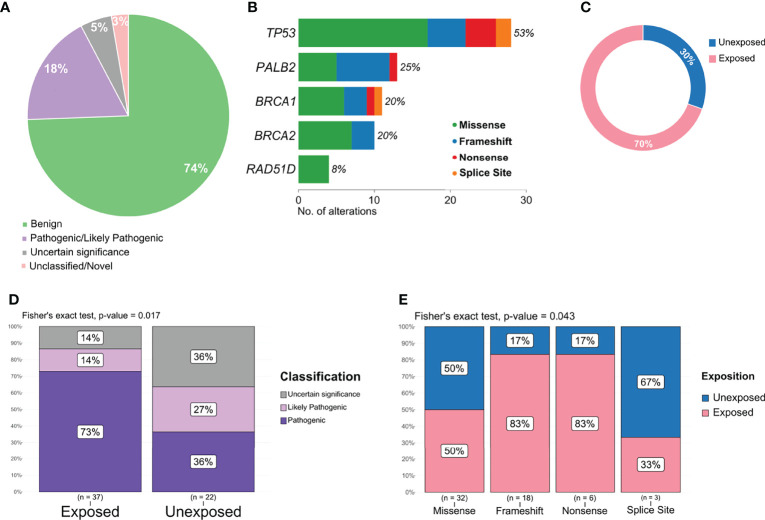
Distribution and frequency of pathogenic, likely pathogenic, and variant of uncertain significance (VUS) variants. **(A)** Graphical representation of variant classification detected in all samples. **(B)** Frequency and type of pathogenic, likely pathogenic, and VUS variant identified in each gene. **(C)** Proportion of pathogenic and likely pathogenic variants detected according to patient exposure status. **(D)** Frequency of variants classified as pathogenic, likely pathogenic, or VUS in exposed and unexposed groups. **(E)** Frequency of variants classified as missense, frameshift, nonsense, and splice site according to exposed and unexposed samples.

Pathogenic or likely pathogenic variants represented 18% of all variants detected (**Figure 4A**) and included missense, frameshift deletions, and nonsense variants, with a high prevalence in *TP53* and *PALB2* (**Figure 4B**). The proportion of pathogenic and/or likely pathogenic variants was higher in the tumor sample of exposed patients than in unexposed patients (**Figure 4C**). When analyzing variants predicted as pathogenic, likely pathogenic, or VUS, exposed patients also presented a significantly higher number than individuals unexposed to pesticides (p = 0.017, **Figure 4D**). We also found a significant difference in the frequency of variant types (missense, splice site, frameshift, and nonsense) in the group of patients exposed or not to pesticides (p = 0.043, **Figure 4E**).

### The Correlation Between Tumor Mutational Burden and Breast Cancer Clinicopathological Parameters and Pesticide Exposure

**Figure 5:**
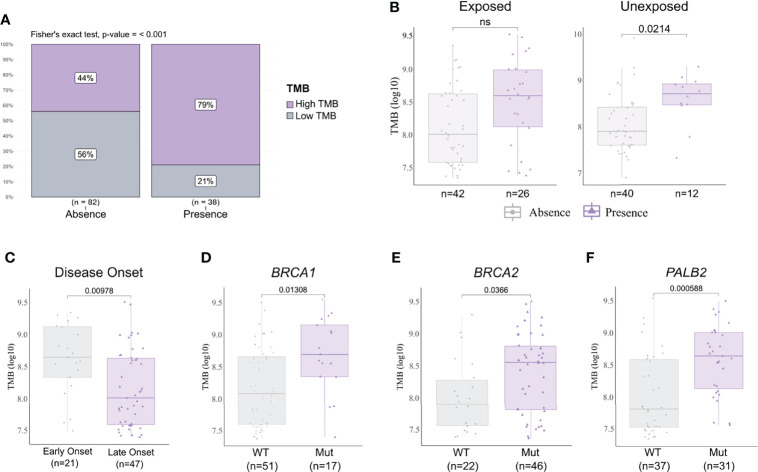
Tumor mutational burden (TMB) and clinicopathological variables according to pesticide exposure. **(A)** Frequency of high and low mutational burden levels in tumor samples with a pathogenic and/or likely pathogenic variant detected or not. Tumors harboring any deleterious variant present increase in mutational burden (p < 0.001). **(B)** TMB levels in samples according to pesticide exposure status and grouped by presence or absence of any predicted pathogenic variant. Tumors from unexposed patients present increased TMB when harboring any deleterious variant (p = 0.0214); this result is not observed in tumors from exposed patients. **(C)** TMB levels in exposed patient samples grouped according to disease onset. We found early-onset tumors (patients with <50 years old) with significantly higher TMB (p = 0.00978) in comparison to late-onset tumors (patients with ≥50 years old). **(D–F)** TMB levels in exposed patient samples grouped according to *BRCA1*, *BRCA2*, and *PALB2* statuses, respectively. Tumors harboring a mutation in *BRCA1* (p = 0.01308), *BRCA2* (p = 0.0366), and/or *PALB2* (p = 0.000588) presented higher TMB than wild-type tumors. The expanded form of “NS” means “No significance”.

We analyzed the mutational burden and its association with different clinicopathological variables and pesticide exposure. In general, samples that presented a pathogenic or likely pathogenic variant had a higher mutational burden (**Figure 5A**). We observed that breast cancer patients exposed to pesticides had no difference regarding mutational burden in the presence or absence of pathogenic variants in the tumor. However, in the unexposed group, tumors harboring any deleterious variant had a higher mutational burden than those with variants of no clinical and/or functional impact (p < 0.02, **Figure 5B**). We also found that only the exposed group of patients diagnosed with breast cancer before 50 years old (p = 0.00978, **Figure 5C**) and patients carrying tumors with *BRCA1* (p = 0.0138, **Figure 5D**), *BRCA2* (p = 0.0366, **Figure 5E**), and/or *PALB2* (p = 0.00058, **Figure 5F**) variants had a higher mutational burden; the same was not observed in the unexposed group (**Supplementary Figure 2**). We found mutational burden increased in tumors that harbor a *TP53* pathogenic or likely pathogenic variant only in unexposed patients (**Supplementary Figure 3**).

## Discussion

Breast cancer tumors and other neoplasias tend to present *TP53* as the most mutated gene (35, 36). However, we found *BRCA2* (~41.5%) with the highest mutational frequency in our study. We highlight that somatic variants of clinical relevance or interest, i.e., classified as pathogenic, likely pathogenic, or VUS, do not usually occur in *BRCA* genes, and we found a slightly higher frequency of *BRCA2* and *BRCA1* (3.1% and 3.5%) variants in comparison to other studies (37–39). Our study also showed the co-occurrence of *BRCA1* and *TP53* mutations in exposed patients, a condition already observed in germline *BRCA1* mutation carriers (40, 41) and found in colorectal cancer tumors, in which co-occurrence of *BRCA1* and *TP53* mutations resulted in poorer prognosis (42). It must be noticed that there are discordances between variant annotations in ClinVar, a large public archive composed mainly of germline variants and the phenotype consequence (43), and COSMIC, the largest public resource of somatic mutations in human cancer (44).

Increased *BRCA1/2* variants are frequent in triple-negative tumors, a tumor type associated with the presence of germline *BRCA1* variants (45). We also detected *BRCA1/2* variants in HER2+, Luminal A, and Luminal B tumors, which indicates an underlying mechanism behind these mutations, but also a publication bias regarding germline variants of triple-negative tumors. It has been demonstrated that environmental exposure to certain toxins can induce haploinsufficiency, a mechanism proposed to contribute to breast cancer development, especially in *BRCA2* cells bearing heterozygous mutations (40, 46). This haploinsufficiency causes the reduction of *BRCA2* function, which sensitizes cells to DNA damage and compromises DNA repair, and under prolonged exposure to these toxins, it can even promote BRCA2 protein depletion in wild-type cells (40). We speculate that pesticide exposure could cause the same effect, as it tends to occur during a long lifetime, not only for tumors bearing deleterious mutations in DNA damage response genes but also in tumors with both functional alleles.

Moreover, there are several studies suggesting the endocrine-disrupting potential of certain pesticides, as reviewed elsewhere (41, 42). This capability enables these substances to mimic and/or antagonize the hormone function. Although current evidence is not fully conclusive of their mechanism of action, exposure to these compounds has been associated with an increased risk of breast cancer (47). We found that exposed patients present an increased frequency of hormone (ER/PR) negative tumors. This result could also be due to the increased presence of *BRCA1/2* mutations and may be indicative of higher genome instability and a decrease in DNA repair, which is in agreement with other studies that show a higher frequency of somatic mutations, mainly in *BRCA2*, in triple-negative tumors (45). Interestingly, it has also been shown that farmers under pesticide exposure that presented chromosomal abnormalities also had lower expression of *BRCA2* (48). This further reinforces that *BRCA2* is essential for DNA stability.

The T>G nucleotide transversion was the most frequent substitution in the exposed patients, whereas unexposed patients presented more T>C substitutions. An Egyptian study found a correlation between T>G and T>C substitutions regarding PIK3CA variants in breast cancer patients and suggested that the oxidative damage accumulation due to age could induce these specific substitutions (49). A high frequency of T>G was found in the presence of oxidized guanine nucleotides (8-oxo-dGTP), an established biomarker of oxidative stress (50). Another study observed that oxidation of the 5-methyl group of thymine generates 5-formyluracil (5-fU), which could induce T>G transversions, has a mutagenic potential, as it could pair wrongly with several bases (51). Another indication that T>G transversions could be associated with oxidative damage and exposure to exogenous substances is the high proportion found in whole-genome sequencing of arsenic exposed lung cancer patients (52). As pesticides increase oxidative stress biomarkers (53) and cause direct DNA damage (54), it is plausible to state that the higher frequency of T>G substitution might be related to the pesticide exposure in this group, as we observe a lower frequency in unexposed patients. As we did not perform whole-exome sequencing (WES), it is impossible to associate our results with any mutational signature for now. However, if we speculate that WES T>G substitutions are predominant in the tumors herein analyzed, it is not a signature associated with any mutational process yet (55, 56).

We found *TP53* variants in 17% of tumors, similar to other breast cancer studies, including one with a Brazilian cohort (36, 57). We identified higher *TP53* missense variants frequency (67.9%, 17/28) than frameshift/nonsense (32.1%, 9/28), with the latter mostly identified in pesticide-exposed patients (77.7%). Two novel splicing variants were identified in canonical splice sites, one in intron 3 (c.96+2T>CTGGT) from an unexposed patient and another in intron 5 (c.559+1G>C) from an exposed patient. Regarding the latter, a variant in this position, but with a different nucleotide change (G>A), was already predicted as pathogenic (58, 59). The missense *TP53* c.578A>G (p.H193R) variant found in two patients (one exposed and one unexposed) in our study was already documented in lung cancer tumors (60). Missense mutations in *TP53* usually produce a stable protein with a significant loss of activity, but frameshift and/or nonsense mutations cause loss of function (61). The nonsense variant c.592G>T (p.E198*) found in two exposed patients causes the premature truncation of the protein and is predicted to result in a loss of the protein function. In summary, all variants were clustered within exons 5–8, the evolutionary conserved DNA-binding region of *TP53* protein, which is considered a hotspot area by the IARC database (62). This indicates that patients under pesticide exposure are more prone to DNA damage in comparison to unexposed ones.

*PALB2* presented the second-highest proportion of pathogenic or likely pathogenic variants in our study. *PALB2* has an important role in cancer development and progression as even heterozygous mutations appear to contribute to early events of oncogenesis (63). We identified two frameshift variants in *PALB2* in seven patients of the exposed group and one nonsense variant, c.1240C>T (p.R414*), in an unexposed patient, also reported in several individuals with HBOC syndrome. The nonsense variant c.1240C>T (p.R414*) and the frameshift c.1314delA (p.F440fs) found in the same sample are localized at the evolutionary conserved chromatin-associated motif (ChAM) domain, which is responsible for *PALB2* chromatin association and DNA repair function (64). It is postulated that variants with strong evidence for pathogenicity in *PALB2* are commonly located in the coiled-coil (CC) motif or the WD40 domain (65). The recurrent frameshift variant found in six patients exposed to pesticides occurs in the *PALB2* c.886delA (p.M296fs) and is localized at the CC motif on the amino-terminal region that mediates *PALB2* interaction with *BRCA1* and *RAD51D.*

All *BRCA1* pathogenic variants were found in exposed patients, except for the c.181T>G (p.C61G), observed in two samples, one from an unexposed patient (**Table 2**). This variant is described as a founder mutation in the Polish population and has already been reported as both germline and somatic origin (66, 67), thus called a “shared variant” (68). This missense mutation occurs in the *BRCA1* RING domain, decreasing BRCA1 availability at DNA damage sites and hindering DNA repair. It has also been demonstrated that *BRCA1^C61G^* mammary tumors develop cisplatin (platinum therapy) resistance, a drug that induces oxidative damage in cells (69). As for *BRCA2* pathogenic variants, three of them were in exposed patients, and only one in an unexposed patient, c.2806_2809delAAAC (p.A938fs), which was already detected in breast, ovary, and lung cancer patients by another study (70).

The increased frequency of pathogenic alterations observed for *TP53*, *PALB2*, and *BRCA1*/*2* in patients under pesticide exposure shown by our results suggests that exposure may have a role in oncogenesis. Mutations in these genes hinder DNA repair, leaving cells more vulnerable to DNA damage and prone to therapy-induced lethality (71) and are also related to therapy resistance mechanisms (69, 72). Somatic mutations may change over time due to selective pressure derived from therapy and genetic instability (72), and the genotoxicity effect of pesticide exposure may also impact this mutational landscape, as several toxic substance exposures produce a characteristic mutational pattern that impaired DNA repair capacity (73–75).

Deleterious variants in DNA damage response genes, mainly in lung cancer, are associated with a higher mutational burden (76), as observed in breast carcinomas with DNA damage repair gene variants (77) and in our tumor samples with *BRCA1*, *BRCA2*, and *PALB2* variants. We found a high mutational burden in patients carrying a pathogenic, likely pathogenic, or VUS variant in both groups analyzed, with the highest frequency of truncating and likely deleterious variants in exposed in comparison to unexposed patients. We found a higher mutational burden in patients exposed to pesticides with early onset of breast cancer (<50 years old) in comparison to patients with late onset (≥50 years old); usually, this finding is expected in older patients (78) due to life accumulation. Evidence suggests that the variation of high mutational burden in cancer types could also be related to chronic mutagenic exposure, i.e., lung cancer patients exposed to tobacco (79). Indeed, individuals exposed to several pesticides have increased DNA damage, including DNA strand breaks, a consequence of the direct exposure and of the oxidative stress generated from it (80–83), which could be the mechanism behind our findings in the exposed group. Interestingly, tumors from unexposed patients harboring TP53 mutations presented significantly higher TMB than those with wild-type tumors; however, this result was not observed in the exposed group. Usually, TP53 mutations are found in tumors with a high mutational burden. For example, in lung cancer, high TMB and TP53 mutations are frequently observed in tumors with the SBS4 signature, which is associated with tobacco smoking (84). This indicates that TP53 mutations are related, at least to some extent, to carcinogen exposure. Still, it has been shown, in a population chronically exposed to pesticides, that there is accumulation of DNA lesions due to low DNA repair activity even at low doses of exposure (85). We found mutations in BRCA1/2 and PALB2 genes to be significantly associated with TMB in exposed patients. Therefore, we presume that these tumors present increased mutational burden due to DNA repair deficiency and thus accumulation of DNA damage without even needing to have impairment of TP53 function.

Regarding an epidemiological overview, a study analyzed the genotoxicity of pesticides approved in the United Kingdom in workers exposed during manufacturing, formulation, or use. The authors reported that, although possible confounding variables were not considered—such as age—and the difficulty to infer causality, there is evidence of an increase in genotoxic biomarkers in pesticide-exposed workers (84). A very recent meta-analysis highlights a significant impact of DNA damage for the pesticide-exposed farmers, regardless of gender, age, pesticide type, or use of personal protective equipment (85). It is important to state that 1/3 of the products recently registered in Brazil contain active substances not approved, or even banned, by the European Commission and that the maximum residue level (i.e., pesticide concentration) considered acceptable in Brazil is higher than that allowed in the United States, Canada, European Union, and other BRICS countries (86). Several Brazilian reports, including one in the same region of our study (87), have described high DNA damage in pesticide-exposed patients. Their results were assessed mainly by cytogenetic analysis and indicate that these individuals are more prone to genetic damage and increased mutation rate (80, 88–90). Moreover, in a population from another Brazilian southern state, global DNA methylation was found to increase in exposed patients in comparison to the unexposed group, a result associated with the inactivation of DNA damage repair genes (91).

## Conclusion

This is the first time a study has shown that occupational exposure to pesticides increases the mutational burden and the mutational status of DNA damage response genes in human breast cancer. Our results reinforce the literature that pesticide exposure causes direct DNA damage. We observed increased mutational burden and deleterious variants in the exposed group, which could be associated primarily with oncogenesis, therapy response, and disease progression. In future studies, an increased observation period should be done in these exposed patients to gather information regarding disease progression and therapeutic response, as our group has not completed a 10-year follow-up yet.

## Data Availability Statement

The original contributions presented in the study are included in the article/**Supplementary Materials**, further inquiries can be directed to the corresponding author/s.

## Ethics Statement

The studies involving human participants were reviewed and approved by Ethics Committees of State University of West Paraná. The patients/participants provided their written informed consent to participate in this study.

## Author Contributions

CRB, CP, and TS conceived the overall study and supervised the research. DR, TS, FMA, and HJ contributed to sample obtention and clinical data collection. RG designed all multiplex PCR assays. CF and TS designed all next-generation sequencing (NGS) experiments. TS and SV performed the experiments. CE, NS, and EA conducted all NGS data processing and somatic variant annotation. MB supervised all the bioinformatics analyses. CE and TS built the figures and performed the statistical analysis, with additional input from all authors. TS, SF, CP, and CRB contributed to the literature search, data analysis, and data interpretation of the results. TS and CRB took the lead in writing the manuscript. All authors provided critical feedback and helped shape the research, analysis, and manuscript. All authors read and approved the final manuscript.

## Funding

This manuscript is financially supported by the following Brazilian Research Agencies: CAPES—Coordenação de Aperfeiçoamento de Pessoal de Nível Superior, CNPq—Conselho Nacional de Desenvolvimento Científico e Tecnológico, Fundação Araucária e Programa de Pesquisa para o SUS (PPSUS), and FAPERJ—Fundação de Amparo à Pesquisa do Estado do Rio de Janeiro. CNPq and FAPERJ supported CB (CNPq 304498/2014-9 and FAPERJ E26/201.200/2014). CNPq/MCTI/FNDCT (402364/2021-0) and CNPq (305335/2021-9) supported CP.

## Conflict of Interest

The authors declare that the research was conducted in the absence of any commercial or financial relationships that could be construed as a potential conflict of interest.

## Publisher’s Note

All claims expressed in this article are solely those of the authors and do not necessarily represent those of their affiliated organizations, or those of the publisher, the editors and the reviewers. Any product that may be evaluated in this article, or claim that may be made by its manufacturer, is not guaranteed or endorsed by the publisher.

## References

[1] Donley N. The USA Lags Behind Other Agricultural Nations in Banning Harmful Pesticides. Environ Heal (2019) 18:44. doi: 10.1186/s12940-019-0488-0 PMC6555703 31170989

[2] Naspolini NF Meyer A Moreira JC Sun H Froes-Asmus CIR Dominguez-Bello MG. Environmental Pollutant Exposure Associated With Altered Early-Life Gut Microbiome: Results From a Birth Cohort Study. Environ Res (2022) 205:112545. doi: 10.1016/j.envres.2021.112545 34896087

[3] Rocha GM Grisolia CK. Why Pesticides With Mutagenic, Carcinogenic and Reproductive Risks Are Registered in Brazil. Dev World Bioeth (2019) 19:148–54. doi: 10.1111/dewb.12211 30520552

[4] Friedrich K Silveira da GR Amazonas JC Gurgel A do M Almeida de VES Sarpa M. Situação Regulatória Internacional De Agrotóxicos Com Uso Autorizado No Brasil: Potencial De Danos Sobre a Saúde E Impactos Ambientais. Cad Saude Publica (2021) 37(4):e00061820. doi: 10.1590/0102-311x00061820 34008735

[5] Pignati WA Lima de S E FAN Lara de SS Correa MLM Barbosa JR Leão LH da C. Distribuição Espacial do Uso De Agrotóxicos No Brasil: Uma Ferramenta Para a Vigilância Em Saúde. Cien Saude Colet (2017) 22:3281–93. doi: 10.1590/1413-812320172210.17742017 29069184

[6] Silva Pinto BG Marques Soares TK Azevedo Linhares M Castilhos Ghisi N. Occupational Exposure to Pesticides: Genetic Danger to Farmworkers and Manufacturing Workers – A Meta-Analytical Review. Sci Total Environ (2020) 748:141382. doi: 10.1016/j.scitotenv.2020.141382 32818891

[7] Viviana Waichman A Eve E Celso da Silva Nina N. Do Farmers Understand the Information Displayed on Pesticide Product Labels? A Key Question to Reduce Pesticides Exposure and Risk of Poisoning in the Brazilian Amazon. Crop Prot (2007) 26:576–83. doi: 10.1016/j.cropro.2006.05.011

[8] Buralli RJ Ribeiro H Leão RS Marques RC Silva DS Guimarães JRD. Conhecimentos, Atitudes E Práticas De Agricultores Familiares Brasileiros Sobre a Exposição Aos Agrotóxicos. Saúde e Soc (2021) 30. doi: 10.1590/s0104-12902021210103

[9] Oliveira Pasiani J Torres P Roniery Silva J Diniz BZ Caldas E. Knowledge, Attitudes, Practices and Biomonitoring of Farmers and Residents Exposed to Pesticides in Brazil. Int J Environ Res Public Health (2012) 9:3051–68. doi: 10.3390/ijerph9093051 PMC3499853 23202670

[10] Hanahan D. Hallmarks of Cancer: New Dimensions. Cancer Discov (2022) 12:31–46. doi: 10.1158/2159-8290.CD-21-1059 35022204

[11] Li X Heyer W-D. Homologous Recombination in DNA Repair and DNA Damage Tolerance. Cell Res (2008) 18:99–113. doi: 10.1038/cr.2008.1 18166982 PMC3087377

[12] Wyman C Kanaar R. DNA Double-Strand Break Repair: All’s Well That Ends Well. Annu Rev Genet (2006) 40:363–83. doi: 10.1146/annurev.genet.40.110405.090451 16895466

[13] Jiang M Jia K Wang L Li W Chen B Liu Y. Alterations of DNA Damage Response Pathway: Biomarker and Therapeutic Strategy for Cancer Immunotherapy. Acta Pharm Sin B (2021) 11:2983–94. doi: 10.1016/j.apsb.2021.01.003 PMC8546664 34729299

[14] Jeggo PA Pearl LH Carr AM. DNA Repair, Genome Stability and Cancer: A Historical Perspective. Nat Rev Cancer (2016) 16:35–42. doi: 10.1038/nrc.2015.4 26667849

[15] Hanahan D Weinberg RA. Hallmarks of Cancer: The Next Generation. Cell (2011) 144:646–74. doi: 10.1016/j.cell.2011.02.013 21376230

[16] van der Groep P. Distinction Between Hereditary and Sporadic Breast Cancer on the Basis of Clinicopathological Data. J Clin Pathol (2006) 59:611–7. doi: 10.1136/jcp.2005.032151 PMC1860390 16603649

[17] Pfeifer GP Denissenko MF Olivier M Tretyakova N Hecht SS Hainaut P. Tobacco Smoke Carcinogens, DNA Damage and P53 Mutations in Smoking-Associated Cancers. Oncogene (2002) 21:7435–51. doi: 10.1038/sj.onc.1205803 12379884

[18] Pike MC Krailo MD Henderson BE Casagrande JT Hoel DG. ‘Hormonal’ Risk Factors, ‘Breast Tissue Age’ and the Age-Incidence of Breast Cancer. Nature (1983) 303:767–70. doi: 10.1038/303767a0 6866078

[19] Anderson KN Schwab RB Martinez ME. Reproductive Risk Factors and Breast Cancer Subtypes: A Review of the Literature. Breast Cancer Res Treat (2014) 144:1–10. doi: 10.1007/s10549-014-2852-7 24477977 PMC4026199

[20] Ruder EH Dorgan JF Kranz S Kris-Etherton PM Hartman TJ. Examining Breast Cancer Growth and Lifestyle Risk Factors: Early Life, Childhood, and Adolescence. Clin Breast Cancer (2008) 8:334–42. doi: 10.3816/CBC.2008.n.038 PMC2666469 18757260

[21] June Yang K Lee J Lui Park H. Organophosphate Pesticide Exposure and Breast Cancer Risk: A Rapid Review of Human, Animal, and Cell-Based Studies. Int J Environ Res Public Health (2020) 17(14):5030. doi: 10.3390/ijerph17145030 PMC7399930 32668751

[22] Niehoff NM Nichols HB White AJ Parks CG D’Aloisio AA Sandler DP. Childhood and Adolescent Pesticide Exposure and Breast Cancer Risk. Epidemiology (2016) 27:326–33. doi: 10.1097/EDE.0000000000000451 PMC4862358 26808595

[23] Panis C Gaboardi SC Kawassaki ACB Dias ECM Teixeira GT Silva da DRP. Characterization of Occupational Exposure to Pesticides and Its Impact on the Health of Rural Women. Rev Ciências Farm Básica e Apl - RCFBA (2022) 43:e748. doi: 10.4322/2179-443X.0748

[24] Gaboardi SC Candiotto LZP Ramos LM. PERFIL DO USO DE AGROTÓXICOS NO SUDOESTE DO PARANÁ (2011 – 2016)/Profile of Pesticides Use in the Southwest of Paraná (2011-2016). Rev NERA (2019) p. 13–40. doi: 10.47946/rnera.v0i46.5566

[25] Gomes R Spinola P da S Brant AC Matta BP Nascimento CM de Aquino Paes SM. Prevalence of Germline Variants in Consensus Moderate-to-High-Risk Predisposition Genes to Hereditary Breast and Ovarian Cancer in BRCA1/2-Negative Brazilian Patients. Breast Cancer Res Treat (2021) 185:851–61. doi: 10.1007/s10549-020-05985-9 33128190

[26] De Armas EM De Miranda Scherer N Lifschitz S Boroni M. Genome Variant Calling Workflow Implementation and Deployment in HPC Infrastructure. In: 2021 IEEE International Conference on Bioinformatics and Biomedicine (BIBM). Houston, TX, USA: IEEE. (2021) p. 1933–40. doi: 10.1109/BIBM52615.2021.9669519

[27] DePristo MA Banks E Poplin R Garimella KV Maguire JR Hartl C. A Framework for Variation Discovery and Genotyping Using Next-Generation DNA Sequencing Data. Nat Genet (2011) 43:491–8. doi: 10.1038/ng.806 PMC3083463 21478889

[28] McLaren W Gil L Hunt SE Riat HS Ritchie GRS Thormann A. The Ensembl Variant Effect Predictor. Genome Biol (2016) 17:122. doi: 10.1186/s13059-016-0974-4 27268795 PMC4893825

[29] Mayakonda A Lin D-C Assenov Y Plass C Koeffler HP. Maftools: Efficient and Comprehensive Analysis of Somatic Variants in Cancer. Genome Res (2018) 28:1747–56. doi: 10.1101/gr.239244.118 PMC6211645 30341162

[30] Wilcoxon F. Individual Comparisons by Ranking Methods. Biom Bull (1945) 1:80. doi: 10.2307/3001968

[31] Fisher RA. “Statistical Methods for Research Workers”. In: Kotz S Johnson NL, editors. Breakthroughs in Statistics: Methodology and Distribution. New York, NY: Springer New York. (1992) p. 66–70. doi: 10.1007/978-1-4612-4380-9_6

[32] Richards S Aziz N Bale S Bick D Das S Gastier-Foster J. Standards and Guidelines for the Interpretation of Sequence Variants: A Joint Consensus Recommendation of the American College of Medical Genetics and Genomics and the Association for Molecular Pathology. Genet Med (2015) 17:405–24. doi: 10.1038/gim.2015.30 PMC4544753 25741868

[33] Shapiro SS Wilk MB. An Analysis of Variance Test for Normality (Complete Samples). Biometrika (1965) 52:591–611. doi: 10.1093/biomet/52.3-4.591

[34] Mann HB Whitney DR. On a Test of Whether One of Two Random Variables Is Stochastically Larger Than the Other. Ann Math Stat (1947) 18:50–60. doi: 10.1214/aoms/1177730491

[35] Cole AJ Zhu Y Dwight T Yu B Dickson K-A Gard GB. Comprehensive Analyses of Somatic TP53 Mutation in Tumors With Variable Mutant Allele Frequency. Sci Data (2017) 4:170120. doi: 10.1038/sdata.2017.120 28872635 PMC5584393

[36] Olivier M Hainaut P. TP53 Mutation Patterns in Breast Cancers: Searching for Clues of Environmental Carcinogenesis. Semin Cancer Biol (2001) 11:353–60. doi: 10.1006/scbi.2001.0390 11562177

[37] Knijnenburg TA Wang L Zimmermann MT Chambwe N Gao GF Cherniack AD. Genomic and Molecular Landscape of DNA Damage Repair Deficiency Across The Cancer Genome Atlas. Cell Rep (2018) 23:239–254.e6. doi: 10.1016/j.celrep.2018.03.076 29617664 PMC5961503

[38] Chen F-M Hou M-F Chang M-Y Wang J-Y Hsieh J-S Ou-yang F. High Frequency of Somatic Missense Mutation of BRCA2 in Female Breast Cancer From Taiwan. Cancer Lett (2005) 220:177–84. doi: 10.1016/j.canlet.2004.10.024 15766593

[39] Kwong A Cheuk IW Shin VY Ho CY Au C-H Ho DN. Somatic Mutation Profiling in BRCA-Negative Breast and Ovarian Cancer Patients by Multigene Panel Sequencing. Am J Cancer Res (2020) 10:2919–32. PMC7539773 33042626

[40] Tan SLW Chadha S Liu Y Gabasova E Perera D Ahmed K. A Class of Environmental and Endogenous Toxins Induces BRCA2 Haploinsufficiency and Genome Instability. Cell (2017) 169:1105–1118.e15. doi: 10.1016/j.cell.2017.05.010 28575672 PMC5457488

[41] Cardona B Rudel RA. US Epa’s Regulatory Pesticide Evaluations Need Clearer Guidelines for Considering Mammary Gland Tumors and Other Mammary Gland Effects. Mol Cell Endocrinol (2020) 518:110927. doi: 10.1016/j.mce.2020.110927 32645345 PMC9183204

[42] Bretveld RW Thomas CM Scheepers PT Zielhuis GA Roeleveld N. Pesticide Exposure: The Hormonal Function of the Female Reproductive System Disrupted? Reprod Biol Endocrinol (2006) 4:30. doi: 10.1186/1477-7827-4-30 16737536 PMC1524969

[43] Landrum MJ Lee JM Riley GR Jang W Rubinstein WS Church DM. ClinVar: Public Archive of Relationships Among Sequence Variation and Human Phenotype. Nucleic Acids Res (2014) 42:D980–5. doi: 10.1093/nar/gkt1113 PMC3965032 24234437

[44] Forbes SA Beare D Gunasekaran P Leung K Bindal N Boutselakis H. COSMIC: Exploring the World’s Knowledge of Somatic Mutations in Human Cancer. Nucleic Acids Res (2015) 43:D805–11. doi: 10.1093/nar/gku1075 PMC4383913 25355519

[45] Pop L-A Cojocneanu-Petric R-M Pileczki V Morar-Bolba G Irimie A Lazar V. Genetic Alterations in Sporadic Triple Negative Breast Cancer. Breast (2018) 38:30–8. doi: 10.1016/j.breast.2017.11.006 29202330

[46] Bartek J Lukas J Bartkova J. DNA Damage Response as an Anti-Cancer Barrier: Damage Threshold and the Concept of “Conditional Haploinsufficiency”. Cell Cycle (2007) 6:2344–7. doi: 10.4161/cc.6.19.4754 17700066

[47] Wan MLY Co VA El-Nezami H. Endocrine Disrupting Chemicals and Breast Cancer: A Systematic Review of Epidemiological Studies. Crit Rev Food Sci Nutr (2021), 1–27. doi: 10.1080/10408398.2021.1903382 33819127

[48] Costa MB Farias IR da Silva Monte C Filho LIPF de Paula Borges D de Oliveira RTG. Chromosomal Abnormalities and Dysregulated DNA Repair Gene Expression in Farmers Exposed to Pesticides. Environ Toxicol Pharmacol (2021) 82:103564. doi: 10.1016/j.etap.2020.103564 33326828

[49] Ibrahim IH Abd El-Aziz HG Amer NNL Abd El-Sameea HS. Mutational Pattern of PIK3CA Exon 20 in Circulating DNA in Breast Cancer. Saudi J Biol Sci (2022) 29(4):2828–35. doi: 10.1016/j.sjbs.2022.01.002 PMC9073026 35531214

[50] Christensen S van der Roest B Besselink N Janssen R Boymans S Martens JWM. 5-Fluorouracil Treatment Induces Characteristic T<G Mutations in Human Cancer. Nat Commun (2019) 10:4571. doi: 10.1038/s41467-019-12594-8 31594944 PMC6783534

[51] Kamiya H Murata-Kamiya N Karino N Ueno Y Matsuda A Kasai H. Induction of T → G and T → A Transversions by 5-Formyluracil in Mammalian Cells. Mutat Res Toxicol Environ Mutagen (2002) 513:213–22. doi: 10.1016/S1383-5718(01)00312-6 11719107

[52] Martinez VD Thu KL Vucic EA Hubaux R Adonis M Gil L. Whole-Genome Sequencing Analysis Identifies a Distinctive Mutational Spectrum in an Arsenic-Related Lung Tumor. J Thorac Oncol (2013) 8:1451–5. doi: 10.1097/JTO.0b013e3182a4dd8e 24128716

[53] Hernández AF Lacasaña M Gil F Rodríguez-Barranco M Pla A López-Guarnido O. Evaluation of Pesticide-Induced Oxidative Stress From a Gene–Environment Interaction Perspective. Toxicology (2013) 307:95–102. doi: 10.1016/j.tox.2012.09.007 23032575

[54] Bolognesi C. Genotoxicity of Pesticides: A Review of Human Biomonitoring Studies. Mutat Res Mutat Res (2003) 543:251–72. doi: 10.1016/S1383-5742(03)00015-2 12787816

[55] Nik-Zainal S Morganella S. Mutational Signatures in Breast Cancer: The Problem at the DNA Level. Clin Cancer Res (2017) 23:2617–29. doi: 10.1158/1078-0432.CCR-16-2810 PMC5458139 28572256

[56] Nik-Zainal S Alexandrov LB Wedge DC Van Loo P Greenman CD Raine K. Mutational Processes Molding the Genomes of 21 Breast Cancers. Cell (2012) 149:979–93. doi: 10.1016/j.cell.2012.04.024 PMC3414841 22608084

[57] Simão TA Ribeiro FS Amorim LMF Albano RM Andrada-Serpa MJ Cardoso LEB. TP53 Mutations in Breast Cancer Tumors of Patients From Rio De Janeiro, Brazil: Association With Risk Factors and Tumor Characteristics. Int J Cancer (2002) 101:69–73. doi: 10.1002/ijc.10567 12209590

[58] Andrikopoulou A Terpos E Chatzinikolaou S Apostolidou K Ntanasis-Stathopoulos I Gavriatopoulou M. TP53 Mutations Determined by Targeted NGS in Breast Cancer: A Case-Control Study. Oncotarget (2021) 12:2206–14. doi: 10.18632/oncotarget.28071 PMC8522843 34676052

[59] Li G Guo X Chen M Tang L Jiang H Day JX. Prevalence and Spectrum of AKT1, PIK3CA, PTEN and TP53 Somatic Mutations in Chinese Breast Cancer Patients. PloS One (2018) 13:e0203495. doi: 10.1371/journal.pone.0203495 30212483 PMC6136723

[60] Fathi Z Mousavi SAJ Roudi R Ghazi F. Distribution of KRAS, DDR2, and TP53 Gene Mutations in Lung Cancer: An Analysis of Iranian Patients. PloS One (2018) 13:e0200633. doi: 10.1371/journal.pone.0200633 30048458 PMC6061986

[61] Petitjean A Mathe E Kato S Ishioka C Tavtigian SV Hainaut P. Impact of Mutant P53 Functional Properties on TP53 Mutation Patterns and Tumor Phenotype: Lessons From Recent Developments in the IARC TP53 Database. Hum Mutat (2007) 28:622–9. doi: 10.1002/humu.20495 17311302

[62] Murnyák B Hortobágyi T. Immunohistochemical Correlates of TP53 Somatic Mutations in Cancer. Oncotarget (2016) 7:64910–20. doi: 10.18632/oncotarget.11912 PMC5323125 27626311

[63] Shah SP Morin RD Khattra J Prentice L Pugh T Burleigh A. Mutational Evolution in a Lobular Breast Tumour Profiled at Single Nucleotide Resolution. Nature (2009) 461:809–13. doi: 10.1038/nature08489 19812674

[64] Bleuyard J Buisson R Masson J Esashi F. ChAM, a Novel Motif That Mediates PALB2 Intrinsic Chromatin Binding and Facilitates DNA Repair. EMBO Rep (2012) 13:135–41. doi: 10.1038/embor.2011.243 PMC3271335 22193777

[65] Nepomuceno TC Carvalho MA Rodrigue A Simard J Masson J-Y Monteiro ANA. PALB2 Variants: Protein Domains and Cancer Susceptibility. Trends Cancer (2021) 7:188–97. doi: 10.1016/j.trecan.2020.10.002 33139182

[66] Ratajska M Koczkowska M Żuk M Gorczyński A Kuźniacka A Stukan M. Detection of BRCA1/2 Mutations in Circulating Tumor DNA From Patients With Ovarian Cancer. Oncotarget (2017) 8:101325–32. doi: 10.18632/oncotarget.20722 PMC5731877 29254167

[67] Nguyen-Dumont T Karpinski P Sasiadek MM Akopyan H Steen JA Theys D. Genetic Testing in Poland and Ukraine: Should Comprehensive Germline Testing of BRCA1 and BRCA2 Be Recommended for Women With Breast and Ovarian Cancer? Genet Res (Camb) (2020) 102:e6. doi: 10.1017/S0016672320000075 32772980 PMC7443769

[68] Meyerson W Leisman J Navarro FCP Gerstein M. Origins and Characterization of Variants Shared Between Databases of Somatic and Germline Human Mutations. BMC Bioinf (2020) 21:227. doi: 10.1186/s12859-020-3508-8 PMC7273669 32498674

[69] Drost R Bouwman P Rottenberg S Boon U Schut E Klarenbeek S. BRCA1 RING Function Is Essential for Tumor Suppression But Dispensable for Therapy Resistance. Cancer Cell (2011) 20:797–809. doi: 10.1016/j.ccr.2011.11.014 22172724

[70] Xu Z Wang Y Wang L Cui F Zhang L Xiong J. Characteristics of BRCA1/2 Pathogenic Germline Mutations in Chinese NSCLC Patients and a Comparison With HBOC. Hered Cancer Clin Pract (2021) 19:16. doi: 10.1186/s13053-021-00174-1 33563323 PMC7871612

[71] Janatova M Zikan M Dundr P Matous B Pohlreich P. Novel Somatic Mutations in the BRCA1 Gene in Sporadic Breast Tumors. Hum Mutat (2005) 25:319–9. doi: 10.1002/humu.9308 15712267

[72] Cimadamore A Lopez-Beltran A Massari F Santoni M Mazzucchelli R Scarpelli M. Germline and Somatic Mutations in Prostate Cancer: Focus on Defective DNA Repair, PARP Inhibitors and Immunotherapy. Futur Oncol (2020) 16:75–80. doi: 10.2217/fon-2019-0745 31916449

[73] Tan HW Liang Z-L Yao Y Wu D-D Mo H-Y Gu J. Lasting DNA Damage and Aberrant DNA Repair Gene Expression Profile Are Associated With Post-Chronic Cadmium Exposure in Human Bronchial Epithelial Cells. Cells (2019) 8:842. doi: 10.3390/cells8080842 PMC6721754 31390735

[74] Poon S McPherson JR Tan P Teh B Rozen SG. Mutation Signatures of Carcinogen Exposure: Genome-Wide Detection and New Opportunities for Cancer Prevention. Genome Med (2014) 6:24. doi: 10.1186/gm541 25031618 PMC4062065

[75] Weng M Lee H-W Park S-H Hu Y Wang H-T Chen L-C. Aldehydes are the Predominant Forces Inducing DNA Damage and Inhibiting DNA Repair in Tobacco Smoke Carcinogenesis. Proc Natl Acad Sci (2018) 115:E6152–61. doi: 10.1073/pnas.1804869115 PMC6142211 29915082

[76] Dai J Jiang M He K Wang H Chen P Guo H. DNA Damage Response and Repair Gene Alterations Increase Tumor Mutational Burden and Promote Poor Prognosis of Advanced Lung Cancer. Front Oncol (2021) 11:708294. doi: 10.3389/fonc.2021.708294 34604048 PMC8479169

[77] Mei P Freitag CE Wei L Zhang Y Parwani AV Li Z. High Tumor Mutation Burden is Associated With DNA Damage Repair Gene Mutation in Breast Carcinomas. Diagn Pathol (2020) 15:50. doi: 10.1186/s13000-020-00971-7 32393302 PMC7212599

[78] Chalmers ZR Connelly CF Fabrizio D Gay L Ali SM Ennis R. Analysis of 100,000 Human Cancer Genomes Reveals the Landscape of Tumor Mutational Burden. Genome Med (2017) 9:34. doi: 10.1186/s13073-017-0424-2 28420421 PMC5395719

[79] Shao C Li G Huang L Pruitt S Castellanos E Frampton G. Prevalence of High Tumor Mutational Burden and Association With Survival in Patients With Less Common Solid Tumors. JAMA Netw Open (2020) 3:e2025109. doi: 10.1001/jamanetworkopen.2020.25109 33119110 PMC7596577

[80] Hilgert Jacobsen-Pereira C dos Santos CR Troina Maraslis F Pimentel L Feijó AJL Iomara Silva C. Markers of Genotoxicity and Oxidative Stress in Farmers Exposed to Pesticides. Ecotoxicol Environ Saf (2018) 148:177–83. doi: 10.1016/j.ecoenv.2017.10.004 29055201

[81] Kapeleka JA Sauli E Ndakidemi PA. Pesticide Exposure and Genotoxic Effects as Measured by DNA Damage and Human Monitoring Biomarkers. Int J Environ Health Res (2021) 31:805–22. doi: 10.1080/09603123.2019.1690132 31736325

[82] Barrón Cuenca J Tirado N Barral J Ali I Levi M Stenius U. Increased Levels of Genotoxic Damage in a Bolivian Agricultural Population Exposed to Mixtures of Pesticides. Sci Total Environ (2019) 695:133942. doi: 10.1016/j.scitotenv.2019.133942 31756860

[83] Doğanlar ZB Doğanlar O Tozkir H Gökalp FD Doğan A Yamaç F. Nonoccupational Exposure of Agricultural Area Residents to Pesticides: Pesticide Accumulation and Evaluation of Genotoxicity. Arch Environ Contam Toxicol (2018) 75:530–44. doi: 10.1007/s00244-018-0545-7 30003277

[84] Bull S Fletcher K Boobis AR Battershill JM. Evidence for Genotoxicity of Pesticides in Pesticide Applicators: A Review. Mutagenesis (2006) 21:93–103. doi: 10.1093/mutage/gel011 16567350

[85] Nascimento F de A Silva D de M Pedroso TMA Ramos JSA Parise MR. Farmers Exposed to Pesticides Have Almost Five Times More DNA Damage: A Meta-Analysis Study. Environ Sci Pollut Res (2022) 29:805–16. doi: 10.1007/s11356-021-15573-z 34342827

[86] Braga ARC de Rosso VV Harayashiki CAY Jimenez PC Castro ÍB. Global Health Risks From Pesticide Use in Brazil. Nat Food (2020) 1:312–4. doi: 10.1038/s43016-020-0100-3 37128098

[87] Marcelino A Wachtel C Ghisi N. Are Our Farm Workers in Danger? Genetic Damage in Farmers Exposed to Pesticides. Int J Environ Res Public Health (2019) 16:358. doi: 10.3390/ijerph16030358 PMC6388205 30691246

[88] Bernieri T Moraes MF Ardenghi PG Basso da Silva L. Assessment of DNA Damage and Cholinesterase Activity in Soybean Farmers in Southern Brazil: High Versus Low Pesticide Exposure. J Environ Sci Heal Part B (2020) 55:355–60. doi: 10.1080/03601234.2019.1704608 31868080

[89] Silvério ACP Machado SC Azevedo L Nogueira DA de Castro Graciano MM Simões JS. Assessment of Exposure to Pesticides in Rural Workers in Southern of Minas Gerais, Brazil. Environ Toxicol Pharmacol (2017) 55:99–106. doi: 10.1016/j.etap.2017.08.013 28843102

[90] Tomiazzi JS Judai MA Nai GA Pereira DR Antunes PA Favareto APA. Evaluation of Genotoxic Effects in Brazilian Agricultural Workers Exposed to Pesticides and Cigarette Smoke Using Machine-Learning Algorithms. Environ Sci pollut Res (2018) 25:1259–69. doi: 10.1007/s11356-017-0496-y 29086360

[91] Benedetti D Lopes Alderete B de Souza CT Ferraz Dias J Niekraszewicz L Cappetta M. DNA Damage and Epigenetic Alteration in Soybean Farmers Exposed to Complex Mixture of Pesticides. Mutagenesis (2018) 33:87–95. doi: 10.1093/mutage/gex035 29244183

