# Corrigendum: Somatic DNA damage response and homologous repair gene alterations and its association with tumor variant burden in breast cancer patients with occupational exposure to pesticides

**DOI:** 10.3389/fonc.2022.1014392

**Published:** 2022-09-23

**Authors:** Thalita Basso Scandolara, Sara Ferreira Valle, Cristiane Esteves, Nicole de Miranda Scherer, Elvismary M. de Armas, Carolina Furtado, Renan Gomes, Mariana Boroni, Hellen dos Santos Jaques, Fernanda Mara Alves, Daniel Rech, Carolina Panis, Cibele Rodrigues Bonvicino

**Affiliations:** ^1^ Department of Genetics, Biology Institute, Universidade Federal do Rio de Janeiro, Rio de Janeiro, Brazil; ^2^ Bioinformatics and Computational Biology Laboratory, Instituto Nacional de Câncer José Alencar Gomes da Silva (INCA), Rio de Janeiro, Brazil; ^3^ Department of Informatics, Pontificia Universidade Católica (PUC)-Rioo, Rio de Janeiro, Brazil; ^4^ Division of Genetics, Instituto Nacional de Câncer José Alencar Gomes da Silva (INCA), Rio de Janeiro, Brazil; ^5^ Laboratory of Tumor Biology, State University of West Parana, Francisco Beltrão, Brazil; ^6^ Francisco Beltrão Cancer Hospital, Francisco Beltrão, Brazil

**Keywords:** breast cancer, pesticides, occupational exposure, mutational burden, somatic

In the published article, there was an error in the author list, and author Dr. Renan G. Gomes Júnior was erroneously excluded. The corrected author list appears below.

Thalita Basso Scandolara^1^, Sara Ferreira Valle^1^, Cristiane Esteves^2^, Nicole de Miranda Scherer^2^, Elvismary M. de Armas^2,3^, Carolina Furtado^4^, Renan Gomes^4^, Mariana Boroni^2^, Hellen dos Santos Jaques^5^, Fernanda Mara Alves^5^, Daniel Rech^5,6^, Carolina Panis^5^, Cibele Rodrigues Bonvicino^1,4^*

## Author Contributions

CRB, CP and TBS conceived the overall study and supervised the research. DR, TBS, FMV and HSJ contributed to sample obtention and clinical data collection. **RG designed all multiplex PCR assays**. CF and TBS designed all NGS experiments. TBS and SFV performed the experiments. CE, NMS and EMA conducted all NGS data processing and somatic variant annotation. MB supervised all the bioinformatic analysis. CE and TBS built the figures and performed the statistical analysis, with additional input from all authors. TBS, SFV, CP and CRB contributed to the literature search, data analysis and data interpretation of the results. TBS and CRB took the lead in writing the manuscript. All authors provided critical feedback and helped shape the research, analysis, and manuscript.

The authors apologize for this error and state that this does not change the scientific conclusions of the article in any way. The original article has been updated.

## Publisher’s note

All claims expressed in this article are solely those of the authors and do not necessarily represent those of their affiliated organizations, or those of the publisher, the editors and the reviewers. Any product that may be evaluated in this article, or claim that may be made by its manufacturer, is not guaranteed or endorsed by the publisher.

