# Supplementary material for: Somatic DNA Damage Response and Homologous Repair Gene Alterations and Its Association With Tumor Variant Burden in Breast Cancer Patients With Occupational Exposure to Pesticides
Source: Front Oncol. 2022 Jul 8;12:904813. doi: 10.3389/fonc.2022.904813 (PMC9305859; doi:10.3389/fonc.2022.904813)
Supplement: Supplementary file 1 [file DataSheet_1.pdf]

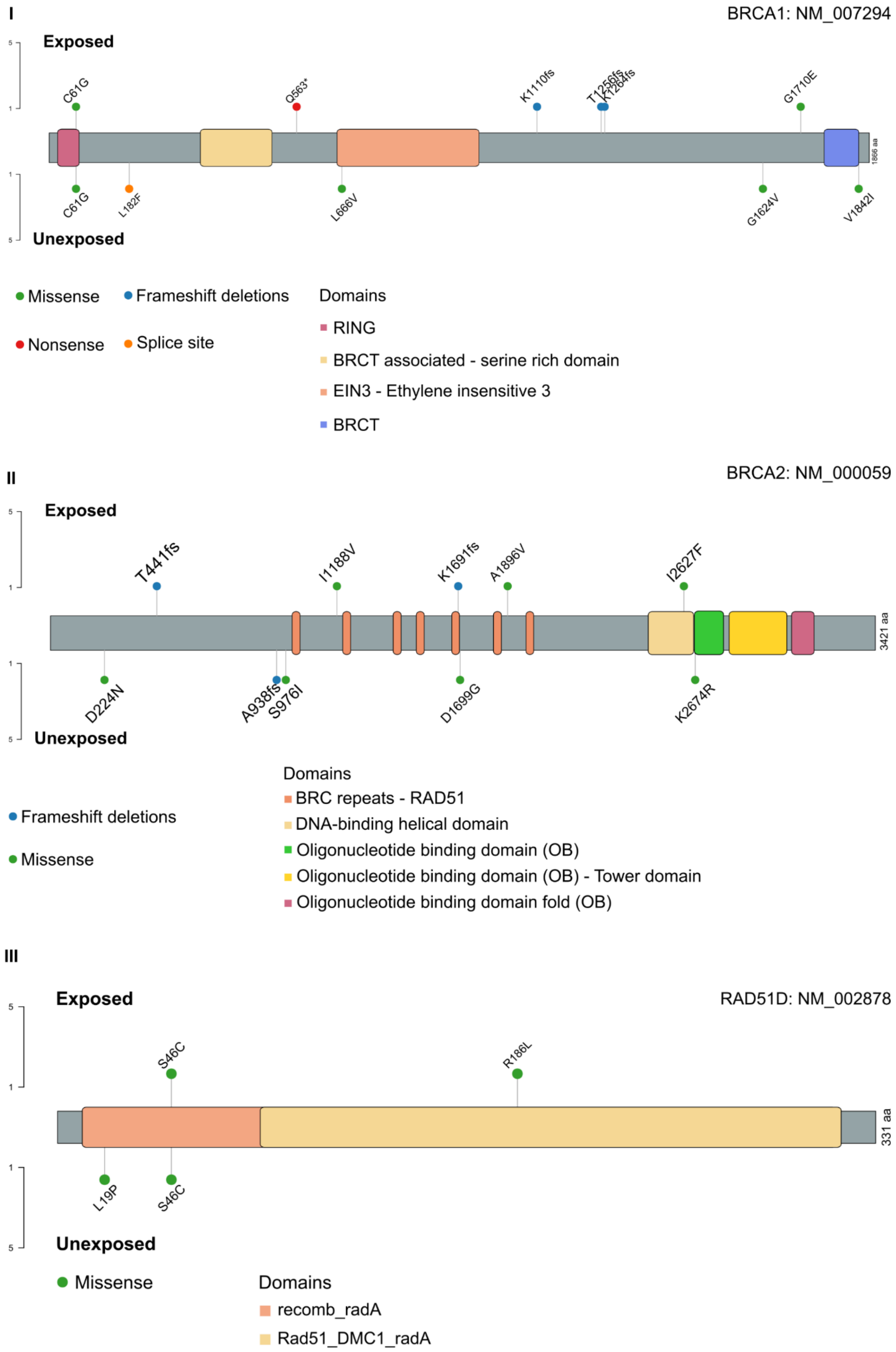
I
BRCA1: NM_007294
Exposed
C61G
Q563*
K1110fs
G1710E
C61G
L182F
L666V
G1624V
V1842I
Unexposed
Missense
Frameshift deletions
Nonsense
Splice site
Domains
RING
BRCT associated - serine rich domain
EIN3 - Ethylene insensitive 3
BRCT
II
BRCA2: NM_000059
Exposed
T441fs
I1188V
K1691fs
A1896V
I2627F
3421 aa
D224N
A938fs
S976I
D1699G
K2674R
Unexposed
Domains
BRC repeats - RAD51
DNA-binding helical domain
Oligonucleotide binding domain (OB)
Oligonucleotide binding domain (OB) - Tower domain
Oligonucleotide binding domain fold (OB)
Frameshift deletions
Missense
III
Exposed
RAD51D: NM_002878
S46C
R186L
331 aa
L19P
S46C
Unexposed
Missense
Domains
recomb_radA
Rad51_DMC1_radA

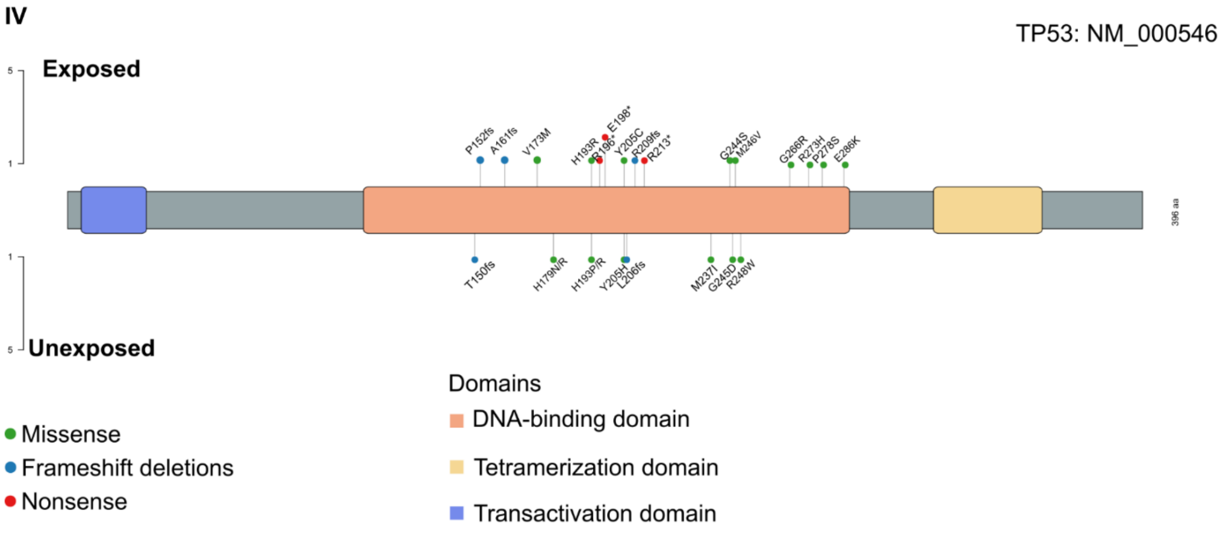

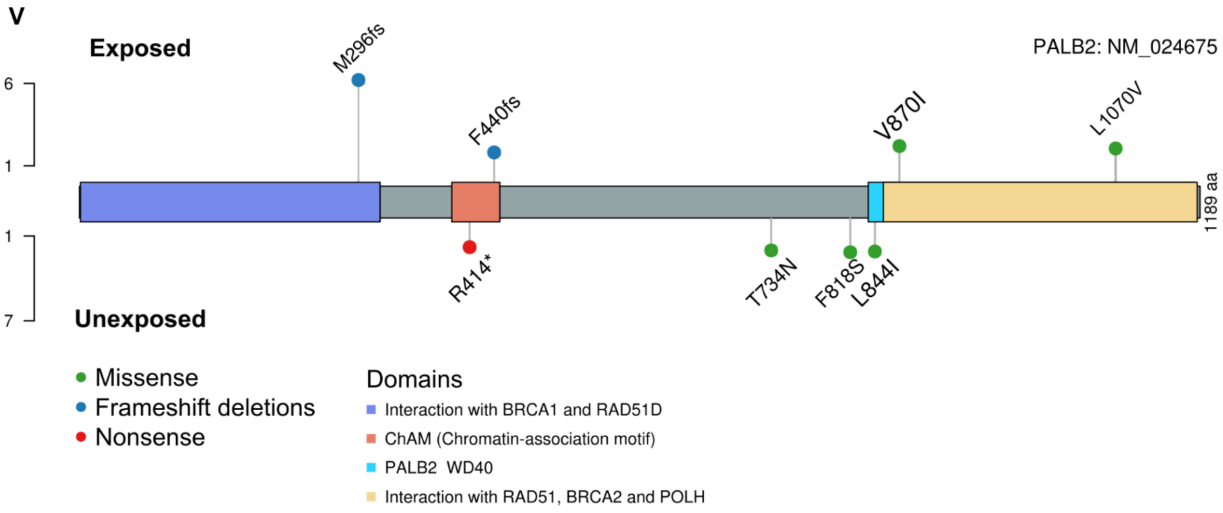

Supplementary Figure 1. Lollipop plot highlighting pathogenic, likely pathogenic and VUS variants found in *BRCA1* (I), *BRCA2* (II), *RAD51D* (III), *TP53* (IV) and *PALB2* (V) genes.

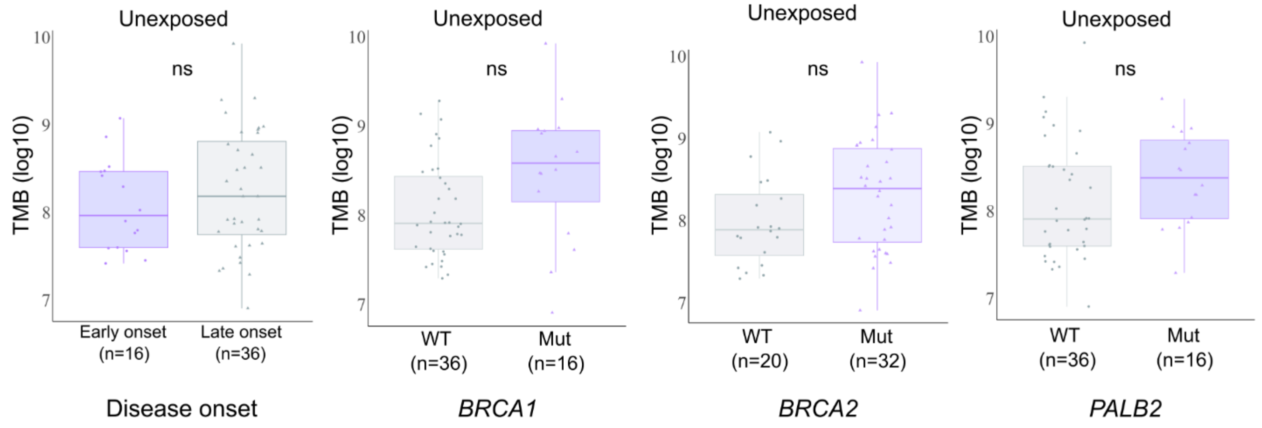

Supplementary Figure 2. Tumor mutational burden levels in unexposed patients according to disease onset (**a**, early onset <50 years old and late onset ≥50 years old), and gene mutation status of *BRCA1* (**b**), *BRCA2* (**c**), and *PALB2* (**d**). WT = wild-type, Mut = mutated, ns = no significance.

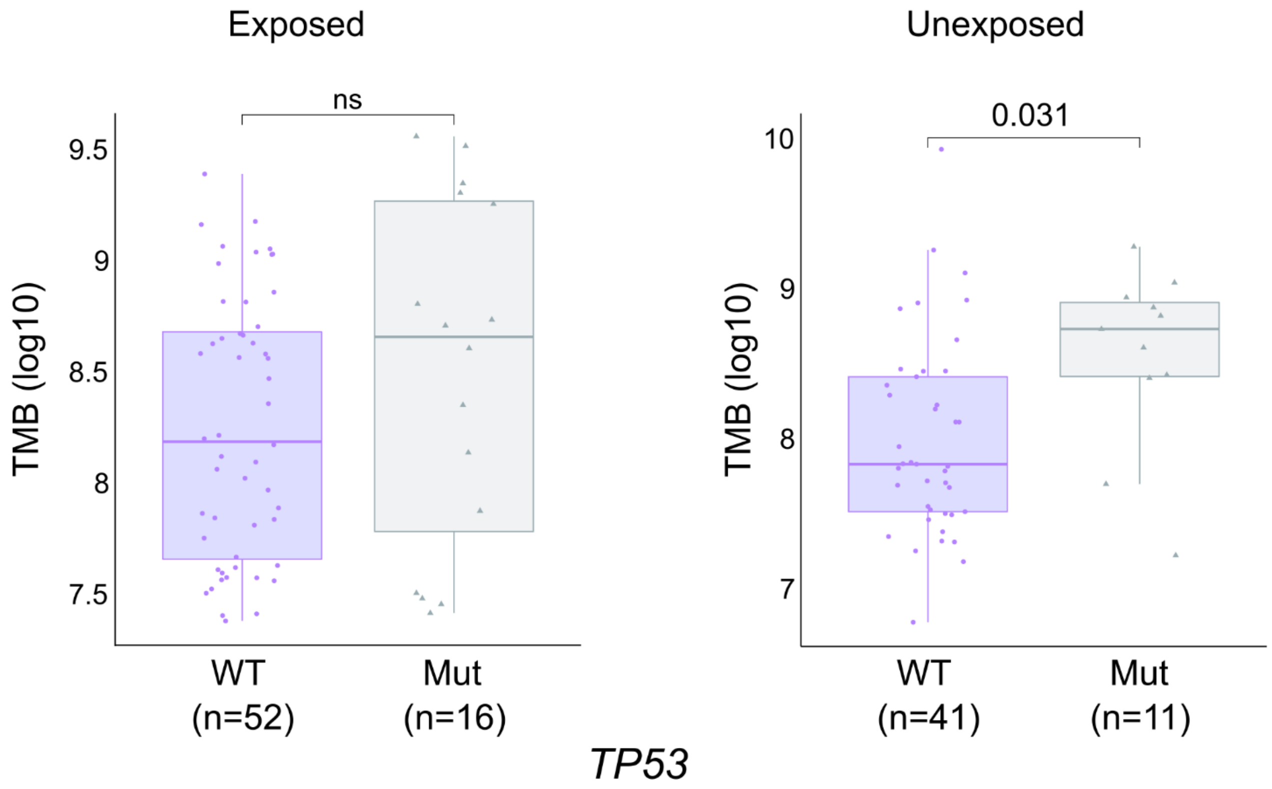

Supplementary Figure 3. Tumor mutational burden levels according to *TP53* status in exposed and unexposed patients. Tumors from unexposed patients with a *TP53* mutation present higher TMB in comparison to WT tumors. This difference was not observed in tumors from exposed patients. WT = wild-type, Mut = mutated, ns = no significance.
