# Supplementary material for: Somatic DNA Damage Response and Homologous Repair Gene Alterations and Its Association With Tumor Variant Burden in Breast Cancer Patients With Occupational Exposure to Pesticides
Source: Front Oncol. 2022 Jul 8;12:904813. doi: 10.3389/fonc.2022.904813 (PMC9305859; doi:10.3389/fonc.2022.904813)
Supplement: Supplementary file 2 [file Table_1.docx]

**Supplementary Table 1.** Tumor mutational burden (TMB) levels grouped by the median-value into high and low TMB.

| Sample Number | Mutational Burden (log scale) | TMB | Pesticide Exposure |
| --- | --- | --- | --- |
| 16 | 7.985095 | Low TMB | Unexposed |
| 18 | 8.142187 | High TMB | Exposed |
| 21 | 8.142187 | High TMB | Unexposed |
| 25 | 8.573764 | High TMB | Exposed |
| 27 | 7.910149 | Low TMB | Exposed |
| 31 | 7.963308 | Low TMB | Exposed |
| 32 | 8.44255 | High TMB | Unexposed |
| 33 | 9.465233 | High TMB | Exposed |
| 38 | 9.00062 | High TMB | Exposed |
| 39 | 7.380964 | Low TMB | Unexposed |
| 48 | 8.976587 | High TMB | Exposed |
| 49 | 8.595155 | High TMB | Exposed |
| 52 | 7.859673 | Low TMB | Unexposed |
| 53 | 8.377431 | High TMB | Unexposed |
| 54 | 7.569404 | Low TMB | Unexposed |
| 55 | 7.765777 | Low TMB | Unexposed |
| 57 | 7.883265 | Low TMB | Unexposed |
| 59 | 7.872711 | Low TMB | Unexposed |
| 62 | 7.829059 | Low TMB | Unexposed |
| 63 | 7.37616 | Low TMB | Unexposed |
| 64 | 7.535115 | Low TMB | Exposed |
| 73 | 8.224524 | High TMB | Unexposed |
| 76 | 7.514725 | Low TMB | Exposed |
| 79 | 7.550209 | Low TMB | Unexposed |
| 80 | 7.343164 | Low TMB | Exposed |
| 98 | 9.338174 | High TMB | Exposed |
| 111 | 8.804875 | High TMB | Exposed |
| 119 | 7.569404 | Low TMB | Exposed |
| 122 | 9.883685 | High TMB | Unexposed |
| 127 | 7.290481 | Low TMB | Unexposed |
| 138 | 8.00437 | Low TMB | Exposed |
| 142 | 9.296443 | High TMB | Exposed |
| 143 | 8.468403 | High TMB | Unexposed |
| 144 | 8.759265 | High TMB | Exposed |
| 150 | 8.820553 | High TMB | Unexposed |
| 152 | 7.875339 | Low TMB | Unexposed |
| 156 | 8.142187 | High TMB | Unexposed |
| 159 | 8.466817 | High TMB | Unexposed |
| 162 | 8.736179 | High TMB | Unexposed |
| 170 | 7.77764 | Low TMB | Exposed |
| 172 | 7.737868 | Low TMB | Unexposed |
| 176 | 9.508073 | High TMB | Exposed |
| 178 | 8.934604 | High TMB | Exposed |
| 186 | 8.25099 | High TMB | Unexposed |
| 187 | 9.2408 | High TMB | Unexposed |
| 188 | 7.420249 | Low TMB | Exposed |
| 198 | 7.754054 | Low TMB | Unexposed |
| 201 | 7.751725 | Low TMB | Exposed |
| 203 | 8.314252 | High TMB | Unexposed |
| 212 | 7.829059 | Low TMB | Exposed |
| 213 | 8.480379 | High TMB | Unexposed |
| 225 | 7.518401 | Low TMB | Unexposed |
| 227 | 8.873868 | High TMB | Unexposed |
| 229 | 9.094782 | High TMB | Unexposed |
| 240 | 7.815334 | Low TMB | Exposed |
| 241 | 9.203696 | High TMB | Exposed |
| 248 | 7.804243 | Low TMB | Exposed |
| 251 | 8.937772 | High TMB | Unexposed |
| 256 | 6.863738 | Low TMB | Unexposed |
| 259 | 9.032194 | High TMB | Unexposed |
| 260 | 8.921409 | High TMB | Unexposed |
| 271 | 8.505594 | High TMB | Exposed |
| 274 | 8.609674 | High TMB | Exposed |
| 277 | 8.29405 | High TMB | Exposed |
| 283 | 7.443899 | Low TMB | Exposed |
| 284 | 8.985598 | High TMB | Exposed |
| 285 | 7.500153 | Low TMB | Exposed |
| 286 | 8.036311 | High TMB | Exposed |
| 287 | 9.124163 | High TMB | Exposed |
| 291 | 8.679712 | High TMB | Exposed |
| 297 | 8.648822 | High TMB | Exposed |
| 301 | 8.115067 | High TMB | Exposed |
| 305 | 8.571122 | High TMB | Exposed |
| 308 | 7.443899 | Low TMB | Exposed |
| 313 | 7.318736 | Low TMB | Unexposed |
| 318 | 7.318736 | Low TMB | Exposed |
| 326 | 7.5811 | Low TMB | Unexposed |
| 328 | 7.354042 | Low TMB | Exposed |
| 331 | 8.668016 | High TMB | Unexposed |
| 335 | 8.300068 | High TMB | Exposed |
| 336 | 7.350922 | Low TMB | Exposed |
| 340 | 8.157772 | High TMB | Exposed |
| 343 | 7.250246 | Low TMB | Unexposed |
| 344 | 8.762455 | High TMB | Exposed |
| 346 | 7.846803 | Low TMB | Unexposed |
| 349 | 7.442191 | Low TMB | Unexposed |
| 360 | 8.551526 | High TMB | Exposed |
| 366 | 8.617014 | High TMB | Exposed |
| 367 | 8.078938 | High TMB | Exposed |
| 368 | 8.975268 | High TMB | Exposed |
| 369 | 7.393888 | Low TMB | Exposed |
| 372 | 7.783624 | Low TMB | Exposed |
| 374 | 7.745928 | Low TMB | Unexposed |
| 402 | 7.693018 | Low TMB | Exposed |
| 409 | 7.602904 | Low TMB | Unexposed |
| 414 | 7.724201 | Low TMB | Unexposed |
| 428 | 8.061352 | High TMB | Exposed |
| 430 | 8.902856 | High TMB | Unexposed |
| 431 | 8.527244 | High TMB | Exposed |
| 432 | 9.253248 | High TMB | Exposed |
| 437 | 8.866751 | High TMB | Unexposed |
| 462 | 9.110495 | High TMB | Exposed |
| 463 | 8.431015 | High TMB | Unexposed |
| 468 | 8.525561 | High TMB | Exposed |
| 482 | 7.503776 | Low TMB | Exposed |
| 485 | 7.410282 | Low TMB | Unexposed |
| 492 | 7.606921 | Low TMB | Exposed |
| 496 | 7.550209 | Low TMB | Exposed |
| 500 | 8.653586 | High TMB | Exposed |
| 507 | 7.512892 | Low TMB | Exposed |
| 511 | 7.557843 | Low TMB | Unexposed |
| 514 | 8.414336 | High TMB | Exposed |
| 518 | 8.617014 | High TMB | Unexposed |
| 528 | 8.751334 | High TMB | Exposed |
| 530 | 9.01149 | High TMB | Exposed |
| 536 | 7.559761 | Low TMB | Exposed |
| 537 | 9.261634 | High TMB | Unexposed |
| 539 | 8.423399 | High TMB | Unexposed |
| 541 | 7.462881 | Low TMB | Exposed |
| 561 | 8.508894 | High TMB | Exposed |
